# Complications in operative fixation of calcaneal fractures

**DOI:** 10.12669/pjms.324.10225

**Published:** 2016

**Authors:** Ying Li, Rong-Hua Bao, Zhi-Qiang Jiang, Huo-Yan Wu

**Affiliations:** 1 Ying Li, Department of Orthopaedics, Guangdong Hospital of Integrated Traditional and Western Medicine, Foshan 528200, Guangdong, China; 2 Rong-Hua Bao, Department of Orthopaedics, Orthopedics Hospital of Traditional Chinese Medicine of Fuyang, Hangzhou 311400, Zhejiang, China; 3 Zhi-Qiang Jiang, Department of Orthopaedics, Guangdong Hospital of Integrated Traditional and Western Medicine, Foshan 528200, Guangdong, China; 4 Huo-Yan Wu, Department of Orthopaedics, Guangdong Hospital of Integrated Traditional and Western Medicine, Foshan 528200, Guangdong, China

**Keywords:** Associated factors, Calcaneal fractures, Complication, Management

## Abstract

**Objective:**

The purpose of this study focused on a number of factors that have been implicated in calcaneal complications and find the incidence of wound complications.

**Methods:**

This was a retrospective study. A total of 162 patients (176 feet) who underwent calcaneal fractures between 2007 and 2012 were included. The patient’s personal details, age, time from injury to surgery, cause of injury, type of fracture, operative details, operating and tourniquet times were collected from hospital computers and paper records. Evidence of complications including wound infection, wound necrosis, pain, malunion, nonunion, impingement, loss of fixation, ect were studied.

**Results:**

Forty-seven of one hundred and seventy-six fractures (26.704%) had complications, wound infection was noted in seven fractures (3.977%), twelve fractures developed necrosis (6.818%), 14 fractures (7.955%) developed pain. Malunion was found in five fractures (2.841%), nonunion in two fractures (1.136%) and loss of fixation in four fractures (2.272%). Three neurologic injury was also seen in our study (1.705%). Operating time, time from injury to surgery and type of fracture had some association with complications in operative fixation of calcaneal fractures, which showed a statistically significant improvement (P=0.000, 0.031, 0.020, respectively), but there were no evidence that age and tourniquet time affect the incidence of complication after calcaneal fracture surgery (P=0.119, 0.682, respectively).

**Conclusions:**

Despite developments in the surgical treatment of calcaneal fracture, wound complications still remain inevitable. Advanced imaging techniques, less invasive surgical procedures, wealth of anatomical knowledge, surgical experience and better postoperative care should be ensured.

## INTRODUCTION

Calcaneal fractures are the most common fractures of the tarsal bones, representing 2% of all fractures and accounting for 60%-65% of all tarsal fractures.^1^,^2^ Treatment of calcaneal fractures is a considerable challenge due to its unique shape, location and limited soft tissue envelope. While it is becoming more accepted that open reduction and internal fixation is the preferred treatment for calcaneal fractures, Dhillon et al.^3^ considered that open reduction and internal fixation theoretically would provide patients with the possibility of painless weight bearing in daily activities. However, the treatment currently remains controversial because of lower patient satisfaction and higher incidence of complications.

The incidence of post-operative wound complications reported by Sampath et al.^4^ was 30%. Kai et al.^5^ have reported twenty-one fractures (8.79%) involved surgical incision complications, including 8 (3.35%) cases of wound dehiscence, 7 (2.93%) of flap margin necrosis, 5 (2.09%) of hematoma, and one (0.42%) of osteomyelitis. Backes et al.^6^ have reported that a total of 191 patients were included of which 47 patients (24.6%) had a post-operative wound infections; 21 (11.0%) and 26 (13.6%) patients had a superficial and deep wound infection. Other reported incidence has ranged from 10% to 25%, respectively.^7^-^11^ There might be multiple factors influencing the outcome of calcaneal surgical incisions pertinent to the peri-operative period. More studies^12^-^15^ have reported that complications in operative fixation of calcaneal fractures were influenced by the patient’s age, cause and type of fracture, surgical wait time, body mass index, reduction of Böhler’s angle, quality of the suture, skin distraction method, and timing of the suture removal.

The purpose of this study focused on a number of factors that have been implicated in calcaneal complications and find the incidence of wound complications.

## METHODS

This was a retrospective study. A total of 162 patients (176 feet) who underwent calcaneal fractures between 2007 and 2012 were included. This study was conducted in accordance with the declaration of Helsinki after approval from the Ethics Committee of the First Affiliated Hospital of Guangzhou Medical University. Written informed consent was obtained from all participants. The sample consisted of patients who were treated with a calcaneal plate being inserted through a lateral extensile approach during the study period. The patients with associated fractures of the ankle, tarsals, metatarsals, or phalanges were excluded. The patients with open fractures of calcaneal or infection were also excluded.

The patient’s personal details, age, time from injury to surgery, cause of injury, type of fracture, operative details, operating and tourniquet times were collected from hospital computers and paper records. Before reconstructive surgery, conventional radiography of the fractured calcaneus was taken for all cases, and multidetector CT scan was taken to obtain better appreciation of the position and alignment of fracture lines and fracture fragments.

Study included 115 men and 47 women from 21 to 55 years (mean, 35.6 years) of age. The mean follow up after calcaneal reconstruction was 11.4 months (range, 8-14 months). The cause of injury included a fall from a height or motor vehicle accidents. Based on multidetector CT, according to the Sanders classification system,^3^ none of the 176 feet in this study were type I, there were 72 feet of type II fractures (24 type IIA, 31 type IIB and 17 type IIC). 56 feet were type III fractures (17 type IIIAB, 27 type IIIBC and 12 type IIIAC). 48 feet were classified as type IV. Evidence of complications were collected including wound infection (deep infection and superficial infection), wound necrosis, pain (arthritis pain, heel pad pain and diffuse pain), malunion, nonunion, impingement, loss of fixation, ect.

For the purpose of statistical analysis, the sample was divided into complicated and uncomplicated groups. All statistical analyses were performed using the SPSS 16.0 for windows. Smoking, type of fracture, time to surgery, operating and tourniquet times between complicated and uncomplicated groups were tested by Chi-squared tests. *P*<0.05 was defined as statistically significant.

## RESULTS

**Fig.1:**
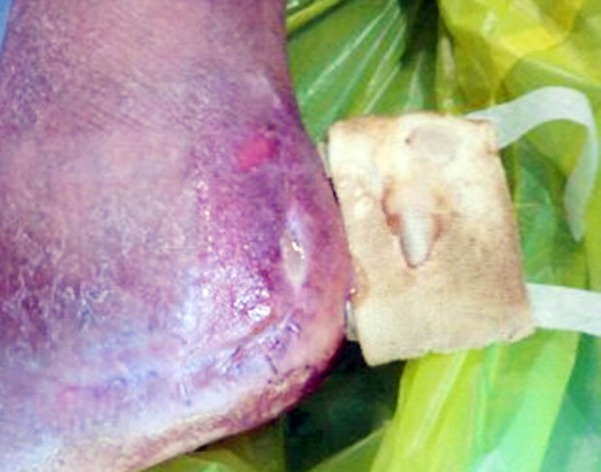
Wound infection in operative fixation of calcaneal fracture.

**Fig.2:**
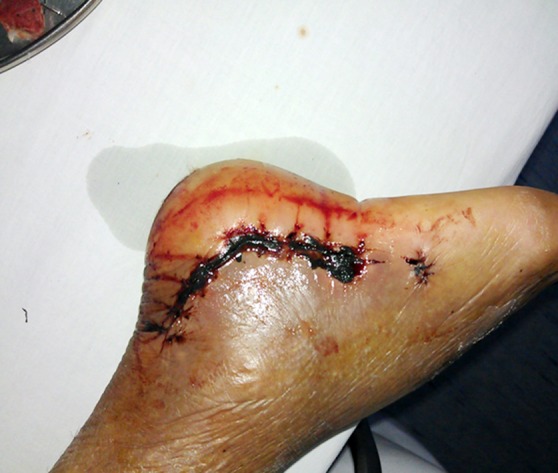
Wound necrosis in operative fixation of calcaneal fracture.

**Fig.3:**
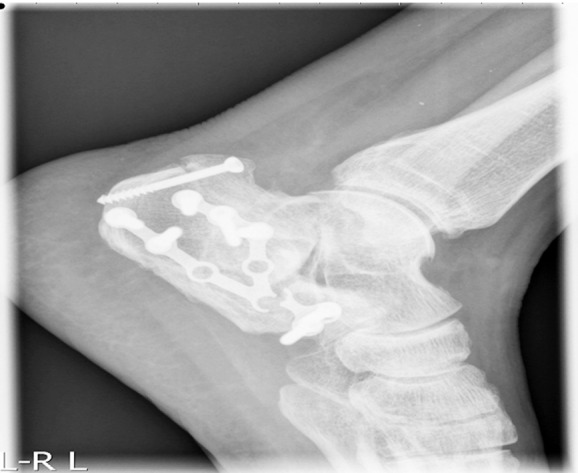
Nail broken in operative fixation of calcaneal fracture.

One hundred and seventy-six closed intra-articular calcaneal fractures were retrospectively assessed. Complication rate in operative fixation of calcaneal fractures is shown in Table-I. Forty-seven of one hundred and seventy-six fractures (26.704%) had complications. Wound infection was noted in seven fractures (3.977%), there were three (1.704%) deep infections and four (2.273%) superficial infections. All of these twelve fractures developed necrosis (6.818%). Pain was reported the most common in 176 fractures, 14 fractures (7.955%) developed pain that included arthritis pain (3 fractures), heel pad pain (5 fractures) and diffuse pain (6 fractures). In the other complications, malunion was found in five fractures (2.841%), nonunion in two fractures (1.136%) and loss of fixation in four fractures (2.272%). three neurologic injury was also seen in our study (1.705%). Although compartment syndrome is a recognized complication, it was not documented in any of our patient’s notes (Fig. 1-3).

**Table-I:** Complication rate in operative fixation of calcaneal fractures.

Variable	Complications	Complication rate (%)
Infection	7(176)	3.977%
Necrosis	12(176)	6.818%
Pain	14(176)	7.955%
Malunion	5(176)	2.841%
Nonunion	2(176)	1.136%
Loss of fixation	4(176)	2.272%
Neurologic injury	3(176)	1.705%

Total	47(176)	26.704%

Relationship between associated factor and complications is shown in Table-II. In operating time, 93 fractures were equal to or less than 120 minutes that 8 fractures developed complications and 83 fractures had more than 120 minutes that 39 fractures developed complications. The difference of them reached statistical significance (*P*=0.000<0.05). The time from injury to surgery, 63 fractures were equal to or less than 1 week that 26 fractures developed complications and 113 fractures were more than one week that 21 fractures developed complications, which showed a statistically significant improvement (*P*=0.031<0.05). In type II, III, and IV of fracture, complications were observed in 12, 16, and 19 fractures respectively, and no complications in 60, 40, and 29 fractures respectively, which showed those to be significant (*P*=0.020<0.05).

**Table-II:** Results of associated factor analysis.

Associated factor	Variable	Complicated group	Uncomplicated group	Total	P
Tourniquet time	≤90 min	20	72	118	0.119
	>90 min	27	57	64	
Operating time	≤120 min	8	85	93	0.000
	>120 min	39	44	83	
Time from injury to surgery	≤1 week	26	48	63	0.031
	>1 week	21	81	113	
Type of fracture	type II	12	60	72	0.020
	type III	16	40	56	
	type IV	19	29	48	
Age	≤35 y	26	70	104	0.682
	>35 y	21	65	72	

Analysis of tourniquet time showed that the 20 fractures which developed complications were equal to or less than 90 minutes and 27 fractures developed complications were more than 90 minutes. Statistical analysis showed no difference in the rate of complications with regard to tourniquet time (*P*=0.119>0.05). This situation also occurs with age (*P*=0.682>0.05). So we were unable to conclude tourniquet time and age as risk factors due to the similarity in both complicated and uncomplicated groups.

## DISCUSSION

Calcaneal is situated at the inferoposterior part of the foot. It plays an important role in walking and running by supporting the axial load from the weight of the body.^16^ Calcaneal fracture is the most common fracture of the tarsal bones. In the Mitchell’s research, the annual incidence of calcaneal fracture was 11.5 per 100,000, and occurred 2.4 times more frequently in males than in females. In males, the incidence was 16.5/100,000/year while that in females was 6.26/100,000/year.^17^ Falls from a height and motor-vehicle accident are the major cause of these large force compression injuries, causing widening of the heel, loss of heel height, and large amounts of articular surface displacement.^18^,^19^ Although not life-threatening, these injuries may result in permanent disability. Open reduction and internal fixation has been established as a standard therapy for calcaneal fractures with good to excellent results. However, as surgical techniques have advanced, there remains uncertainty regarding the complication following operative management of calcaneal fractures.

A fairly high prevalence of complication of calcaneal fractures has been reported. SooHoo et al.^9^ identified 4481 patients who underwent open reduction and internal fixation of their fracture as inpatients within 30 days of the index admission. The short-term rate of complications included a 90-day rate of readmission of 1.03% for wound infection, 0.25% for thromboembolic disease, and 0.22% for mortality. The mid-term rate of subtalar fusion was 3.49% at 5 years post-operatively. Koski et al.^20^ analyzed 126 consecutive patients with 148 operatively treated calcaneal fractures, and wound healing was problematic in 35 cases (24 per cent). Howard et al.^21^ found that there were 226 displaced intra-articular calcaneal fractures with 57 of 226 (25%) fractures having at least one major complication. Compared with these reported studies, our complication rate in operative fixation of calcaneal fractures was up to 26.704%.

Wound infection is the common complication in some previous studies about operative fixation of calcaneal fractures. Svatoš et al.^22^ reported deep wound infection rate of 3.7%. Court-Brown^23^ reported that there were 10 (5.6%) deep infections and 35 (19.7%) superficial infections. Our deep and superficial infection rates are similar to those in recently published series. Wallace^24^ pointed that the management goal for these infections was to prevent direct extension to bone causing osteomyelitis. So radical debridement and aggressive antimicrobial therapy are mandatory in cases of infections, but the plate and screws should be removed if infection is because of lack of effective control. In our study, infections of 6 fractures were cured with radical debridement and aggressive antimicrobial therapy, but 1 fracture plate and screws had to be removed. At the same time we think that soft tissue coverage with local or free flaps has to be considered to avoid protracted courses, shorten operation time and delayed time to surgery can reduce the risk of infection. So we can see that the difference are significant in operation time and complications (*P*=0.000<0.05), time to surgery and complications (*P*=0.031<0.05).

Wound necrosis is the most frequently observed postoperative complication in calcaneal fractures, which is seen in up to 14% of cases after standard osteosynthesis via an extended lateral approach and up to 27% with a bilateral approach.^25^ Our wound necrosis rate of 6.818% is comparable to Popelka’s report that there were 6.25 % superficial marginal wound necrosis.^26^ To avoid wound necrosis, Time to surgery should be delayed until wrinkling of the skin reappears, and the leg must be elevated and cold compresses started on admission in our experience. If wound necrosis has occurred, surgery is inevitable. Cavadas^27^ pointed that for minor necroses (<1.5 cm wide) with supple tissues and no infection, the transverse local subcutaneous flap was effective. For moderate-sized wounds (1.5-5 cm) with no infection, a sural subcutaneous flap was used.

The most common patient complaints are pain. Fourteen fractures (7.955%) developed pain in our study. There are many factors causing the pain. Subtalar incongruity, or penetration of implants into the subtalar joint, or arthritis may lead to pain. We can judge the pain through different parts. Lateral pain is caused by lateral impingement and peroneal tendon pathology, which is characterized by resisted eversion aggravating pain. Anterior pain results primarily from either talar neck impingement or scar tissue in the ankle. Plantar pain secondary to plantar exostosis is the most common, while poorly localized pain is caused by nerve-related problems or complex regional pain syndrome.^28^

Malunion is one associated complication of calcaneal fractures that results in disability. Malunion can affect the function of the surrounding joints and soft tissues. Sequelae include loss of height, heel widening subfibular impingement, calcaneocuboid joint impingement, varus heel, and posttraumatic arthrosis. Infection, malalignment, type of fracture, early weight-bearing can lead to malunion. We find that calcaneal fractures are more serious have more complications, although we do not have statistics on how many cases of malunion occurred in type IV in this study. Identification of the correct source of symptoms is key in selecting the appropriate treatment. Banerjee et al.^29^ considered that early management of calcaneal malunion consisted of nonsurgical methods to improve patient comfort and function. Nonsurgical treatment, such as activity modification, bracing, orthoses, and injection, was effective in many patients. When nonsurgical methods fail, surgery is necessary. The surgical treatments included open reduction, calcaneal osteotomy without subtalar fusion or a reconstruction of calcaneal thalamus and subtalar arthrodesis.^30^

Loss of fixation, nonunion, and neurologic injury are also complications seen with calcaneal fracture reconstruction. Loss of fixation can be prevented with intraoperative fluoroscopy, although patient compliance also plays a role in maintaining fixation.^25^ For neurologic injury, knowledge of anatomy of the calcaneal nerves is necessary to ensure safe surgical intervention in the medial heel region.^31^ Some reports^32^,^33^ have mentioned that the risk of complications increase in smokers and in patients of advanced age, but we have found no evidence that age and tourniquet time affect the incidence of complication after calcaneal fracture surgery.

## CONCLUSIONS

Despite developments in the surgical treatment of calcaneal fracture, wound complications still remain inevitable. In such a controversial and potentially detrimental injury, surgeon should know how to reduce complications. Advanced imaging techniques, less invasive surgical procedures, wealth of anatomical knowledge, surgical experience and better postoperative care should be ensured.
